# Neutralizing Antibodies against California Serogroup Orthobunyaviruses in Human Serum Samples, Montana, USA

**DOI:** 10.3201/eid3104.241520

**Published:** 2025-04

**Authors:** Tyson A. Woods, Stephen F. Johnson, Alyssa B. Evans, Karin E. Peterson

**Affiliations:** Laboratory of Neurological Infections and Immunity, Rocky Mountain Laboratories, National Institute of Allergy and Infectious Diseases, National Institutes of Health, Hamilton, Montana, USA

**Keywords:** Orthobunyaviruses, viruses, meningitis/encephalitis, vector-borne infections, Jamestown Canyon virus, mosquitoborne infections, California serogroup, arboviruses, mosquitoes, Montana, United States

## Abstract

Viral encephalitis is often underreported and undiagnosed. To understand the potential causes of viral encephalitis in the state of Montana, USA, we examined the relative incidence of human infections for the California serogroup (CSG) of Orthobunyaviruses by screening random convenience serum samples obtained from different hospitals across Montana. We initially screened deidentified samples for neutralizing antibodies against Jamestown Canyon virus (JCV), a CSG virus that has caused encephalitis in Montana. We then analyzed JCV-positive samples for neutralization of other CSG viruses, and detected neutralizing antibodies against La Crosse virus, California encephalitis virus, and Trivittatus virus. We also found a high level of cross-reactivity, particularly between JCV and California encephalitis virus. Our findings indicate that the relative CSG virus infection rates in humans are quite high, between 21% and 40%. Clinicians should consider CSG viruses in differential diagnosis for cases of encephalitis of unknown etiology in Montana.

Several arboviruses can invade the central nervous system (CNS) and cause neurologic disease. Diagnosis of the specific arbovirus causing encephalitis often includes measuring neutralizing antibody (NAb) titers to that virus in the blood of patients during and after acute encephalitis. That process can take substantial time and reagents. Thus, understanding which encephalitic viruses are found in different geographic areas is essential for determining potential viruses to examine.

The underreported encephalitic virus, Jamestown Canyon virus (JCV), is a negative sense orthobunyavirus that was first isolated in 1961 (*1*). JCV is a member of the California serogroup (CSG) of genetically and antigenically related mosquitoborne orthobunyaviruses. Although JCV is widely distributed within multiple mosquito species throughout the United States and Canada, human cases of JCV encephalitis were seldom reported before 2013 (*2**–**5*). 

NAbs generated to individual viruses often neutralize other members of the same group (*6*,*7*), which can complicate serologic diagnostics. An IgM test developed for JCV increased the number of detected clinical cases from an average of 3–4 cases per year before 2013 to 40–70 cases per year in the United States after 2013 (*2*–*5*,*8*,*9*). JCV cases have also been misdiagnosed as another member of the CSG, La Crosse virus (LACV), due to cross-reactivity of NAb responses, or misdiagnosed as the unrelated West Nile virus, due to a previous infection (*7*,*10*).

JCV screening utilizes detection of IgM against JCV followed by a plaque reduction neutralization test (PRNT) (*10*,*11*). PRNT has been found to be more reliable than IgM tests for JCV (*12*). Determination of active infection is often confirmed by a 4-fold rise in NAb for JCV via PRNT between blood draws in the acute versus convalescent phases of infection. Similarly, a 4-fold higher NAb titer of one CSG virus compared with other related CSG viruses is considered a positive diagnosis for that virus. If a 4-fold difference between CSG is not reached, then the etiologic agent is classified as a general CSG virus infection. 

Diagnosis of specific CSG viruses can be complicated due to the overlap in locations where viruses are found. For example, in addition to JCV and LACV, 3 other CSG viruses are found in North America and can be associated with neurologic disease in humans. California encephalitis virus (CEV) was discovered in 1943; however, only 4 cases of CEV encephalitis were reported in humans during 1943–2001 (*13*). Trivittatus virus (TVTV) was originally isolated from *Aedes trivittatus* mosquitoes in North Dakota in 1952 and has been associated with at least 1 case of viral encephalitis (*12*,*14*,*15*). Snowshoe hare virus (SSHV) was originally identified in Montana in 1961 and has been associated with several neurologic disease cases in children in North America (*16*–*19*). LACV, originally discovered in La Crosse, Wisconsin, in 1964 and found in Midwest and Appalachian regions of the United States, is a primary cause of pediatric arboviral encephalitis in North America and is associated with ≈70–80 hospitalized cases per year (*11*,*20*–*22*). The cross-reactive NAbs between CSG viruses, and the overlapping host and vector ranges in multiple states and providences, make specific virus identification difficult. Understanding the distribution of CSG viruses, the extent of human infections, and the cross-reactivity of the NAbs would provide vital insights toward determining which viruses should be examined as potential causes of viral encephalitis in different regions in North America.

Most serologic studies on CSG virus infections have been performed in animals (*6*,*23*), and few studies have been performed on human serum. A 1995 study of human serum samples in Sri Lanka that tested for SSHV, TVTV, and Lumbo virus showed NAbs in 9.5% of samples (*24*). A 2016 study of workers in 3 US national parks showed CSG virus antibodies in 28%–36% of samples (*12*). In contrast, NAbs to Cache Valley virus (CVV), a more distantly related orthobunyavirus belonging to the Bunyamwera serogroup, were rarely (3%) detected. That finding was surprising considering CVV is widespread in animals in North America, where it causes spontaneous abortions in cattle and sheep (*25*). However, only 7 cases of CVV encephalitis have been reported in humans (*12*,*25*). We analyzed human viral NAb responses in deidentified human serum samples from across Montana to assess distribution of different CSG viruses in the state. 

## Methods

### Human Serum Samples

**Figure 1:**
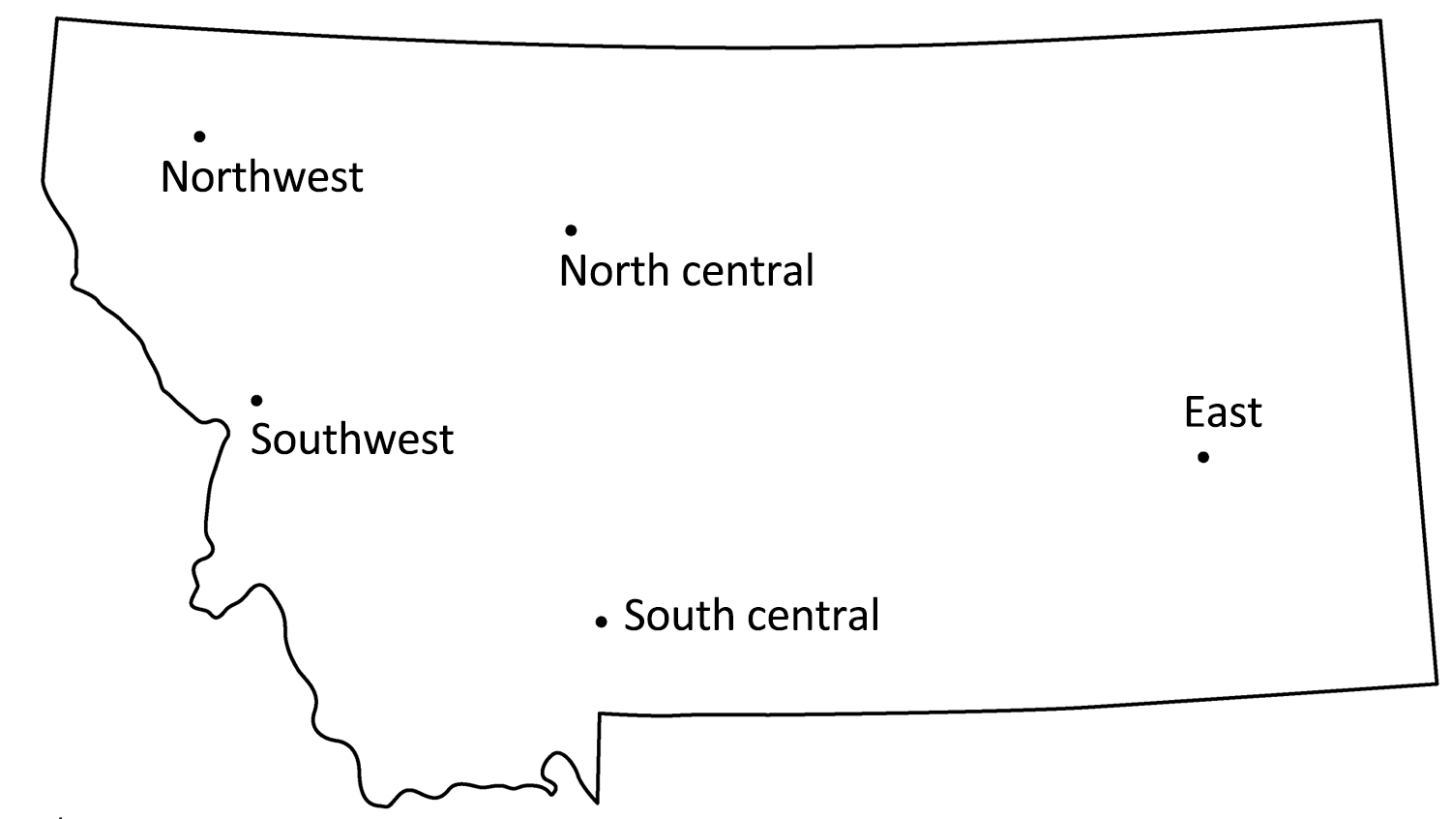
Sampling regions in a study of neutralizing antibodies against California serogroup orthobunyaviruses in human serum samples, Montana, USA. Serum samples were collected from regional hospitals throughout the state.

We obtained convenience deidentified serum samples from hospitals within the Dartmouth Atlas hospital referral regions of Montana (Figure 1). We contacted hospitals by telephone or email, and did not request samples for any specific diseases, symptoms, or signs. Each clinical laboratory provided 100–200 frozen serum samples collected during 2018–2019. Samples arrived frozen at Rocky Mountain Laboratories (RML), National Institutes of Health (Hamilton, MT, USA) and were stored at −20°C until use. Samples were exempt from National Institutes of Health institutional review board approval due to the lack of any personal information other than age and sex. Of 906 samples received, 21 lacked sex or age information. 

### Virus Preparation

Stephen Whitehead (National Institute of Allergy and Infectious Diseases, Bethesda, Maryland, USA) provided LACV (human 1978 strain), and JCV (strain 61V2235). Bob Tesh (University of Texas Medical Branch, Galveston, Texas, USA) provided SSHV(1976) and TVTV (1968). We obtained CVV (stock no. 6V633) and CEV (stock nos. 85–1489) from Brandy Russell (Centers for Disease Control and Prevention, Fort Collins, Colorado, USA). 

We reconstituted lyophilized SSHV and TVTV with Dulbecco’s Modified Eagle Medium (DMEM), 10% fetal bovine serum (FBS), and 1% penicillin-streptomycin (P-S), then filter sterilized and mixed solution with plasmocin to reach ≈27 µg/mL to prevent mycoplasma contamination. We added viruses to cultures of confluent Vero cells for 1 hour at 37°C, then removed. We added fresh media to Vero cells and incubated 6 days. We then used those supernatants to inoculate new Vero cells for 2 days until we observed heavy cytopathic effect. We froze flasks for 24 hours, then thawed and collected and centrifuged lysates before aliquoting supernatants for working virus stocks. We performed the same process for nonlyophilized LACV, CVV, and CEV, excluding reconstitution.

### Mouse Plasma for Virus NAb Determination

We purchased C57BL/6J (B6) mice from Jackson Laboratories (https://www.jax.org) and maintained at RML. For experiments, we intraperitoneally inoculated C57BL/6 mice >6 weeks of age with 200 μL of 10^5^ plaque forming units (PFUs) of virus in phosphate buffered saline, per mouse. We collected blood at 21 days post inoculation using heparin to prevent coagulation. We collected plasma via centrifugation and stored at −80°C until use.

We completed mouse experiments in accordance with National Institutes of Health Guidelines, and experiments were approved by the RML Animal Care and Use Committee. The RML facility is fully accredited by AAALAC International (https://www.aaalac.org). 

### PRNT

We diluted human serum samples or mouse plasma 10-fold, then subsequently performed 5-fold dilutions in DMEM, 2% FBS, 1% P-S. We mixed those samples with 10^2^ PFUs of virus in DMEM/FBS/P-S. We incubated that mixture for 1 hour at 37°C for virus neutralization, then added the mixture to confluent Vero cells and incubated for 1 hour at 37°C. We overlaid 1.5% carboxymethylcellulose in minimum essential medium onto the cells and incubated undisturbed for 5 days. We fixed cells by adding 10% formaldehyde to each well for 1 hour. After fixation, we stained wells with 0.35% crystal violet. 

We determined neutralizing titer by the dilution that inhibited 50% viral PFUs compared with a 10^2^ virus control well. We used a 50% inhibition threshold because it is commonly used for human serum and can be more sensitive in detecting NAb responses than a 90% threshold (*26*–*28*). We ran negative controls with all sets of PRNT assays against each virus.

**Figure 2:**
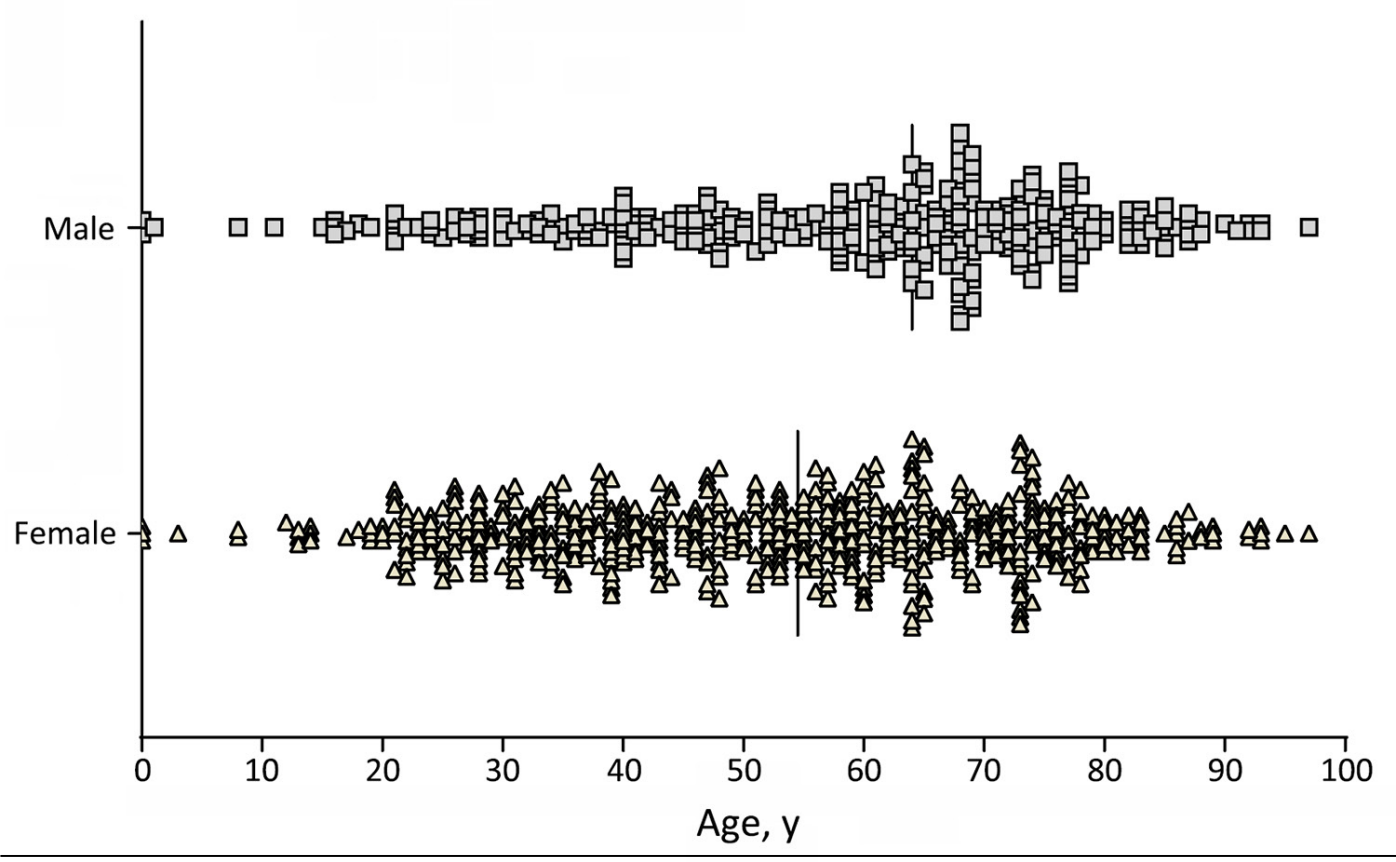
Age and sex distribution of patients whose samples were used in a study of neutralizing antibodies against California serogroup orthobunyaviruses in human serum samples, Montana, USA. Graph shows sample distribution (n = 886; 98%) by sex and age. Vertical bars indicate median age for each sex. Difference in age between sexes was determined by Mann-Whitney test, and p<0.001 was considered statistically significant.

For the initial screen, we analyzed all 906 human serum samples from 6 hospitals for NAb against JCV (Figure 2). For 212 samples from 1 hospital, we did not perform further testing because samples contained insufficient serum. Among the other 694 serum samples, we analyzed JCV-positive samples for CEV, LACV, SSHV, TVTV, and CVV (Figure 2). For the unbiased analysis, we analyzed all 100 samples from the East hospital, regardless of their initial positivity to JCV, for NAb for each virus, i.e., each sample was analyzed independently for LACV, CEV, JCV, SSHV, TVTV, and CVV neutralization.

## Results

### JCV NAb Detection

We obtained 906 deidentified serum samples from 6 hospitals located throughout the state and separated into east, north central, northwest, south central, and southwest regions (Figure 1). We collected 2 sets of samples from the southwest region, which we identified as SW1 and SW2 (Table 1). Of the 906 samples, 885 had sex and age data; 538 samples were from female patients and 347 were from male patients, and median age was 59 years for male patients and 53 years for female patients (Figure 2).

**Table 1:** Positivity of samples tested in a study of neutralizing antibodies against California serogroup orthobunyaviruses in human serum samples, Montana, USA*

Characteristics							Total	Total – SW2	% JCV-positive, n = 159	% Dual positive samples, n = 87
	Hospital region									
	NW	SW1	SW2	NC	SC	E				
Total samples	190	103	212	201	100	100	906	694	NA	NA
JCV-positive	55 (29)	21 (20)	31 (15)	35 (17)	17 (17)	31 (31)	190 (21)	159 (23)	100	NA
Cross-reactivity										
Single high titer										
JCV	18	6	ND	7	4	3	NA	38 (5.4)	23.8	NA
SSHV	ND	ND	ND	ND	ND	ND	NA	0	0	NA
LACV	ND	ND	ND	ND	ND	10	NA	10 (1)	6.2	NA
CEV	4	1	ND	5	4	4	NA	18 (3)	11.3	NA
TVTV	1	2	ND	1		2	NA	6 (1)	3.8	NA
CVV	ND	ND	ND	ND	ND	ND	NA	0	0	NA
Dual or triple high titers†										
Total no.	32	12	ND	22	9	12	NA	87 (12)	54.7	100
JCV + SSHV	ND	ND	ND	ND	ND	ND	NA	0	0	0
JCV + LACV	1	ND	ND	1	ND	1	NA	3 (0.4)	1.9	3.4
JCV + CEV	13	2	ND	8	6	4	NA	33 (4.8)	20.7	37.9
JCV + TVTV	7	ND	ND	7	ND	ND	NA	14 (2)	8.8	16.1
SSHV + LACV	ND	ND	ND	ND	ND	ND	NA	0	0	0
SSHV + CEV	1	ND	ND	1	1	ND	NA	3 (0.4)	1.9	1
LACV + CEV	1	ND	ND	ND	1	ND	NA	2 (0.2)	1.2	2.3
LACV + TVTV	ND	ND	ND	ND	ND	2	NA	2 (0.2)	1.2	2.3
CEV + TVTV	ND	ND	ND	ND	ND	ND	NA	0	0	0
Triple	9	10	ND	5	1	5	NA	30 (4.3)	18.9	34.5

*Values are no. (%) except as indicated. CEV, California encephalitis virus; E, east; JCV, Jamestown Canyon virus; LACV, La Crosse virus; NA, not applicable; NC, north central; ND, not detected; NW, northwest; SC, south central; SSHV, snowshoe hare virus; SW, south-west; TVTV, Trivittatus virus.

**Figure 3:**
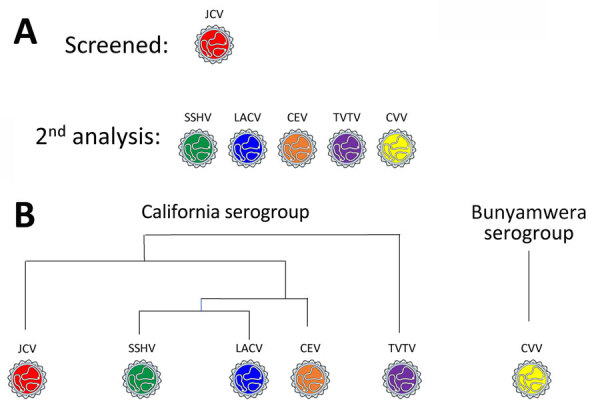
Serum analysis in a study of neutralizing antibodies against California serogroup orthobunyaviruses in human serum samples, Montana, USA. A) Screening process; B) general relationship between analyzed viruses. All samples were initially screened for neutralizing antibody against JCV using a 5-fold dilution series starting at a 1:10 dilution. Samples that were positive for JCV neutralization were subsequently screened for neutralization of other California serogroup viruses and a non–cross-reacting Bunyamwera serogroup virus, CVV. CEV, California encephalitis virus; CVV, Cache Valley virus; JCV, Jamestown Canyon virus; LACV, La Crosse virus; SSHV, snowshoe hare virus; TVTV, Trivittatus virus.

Because JCV was associated with a case of encephalitis in Montana, we initially examined all samples for NAbs against JCV (Figure 3). Of the 906 samples, 190 (21%) were positive for NAb against JCV at >10-fold dilutions (Table 1; Appendix Table 1). The SW2 group had the lowest (15%) percentage of positive samples, and the east group had the highest (31%). Overall, those data indicate that ≈1/5 of patients tested were previously infected with a virus related to JCV, similar to a study of national park workers in Wyoming and Colorado (*12*).

### Cross-Reactivity with Other CSG Viruses

We analyzed JCV-positive samples for neutralization of other CSG viruses found in North America. We did not test the 31 JCV-positive samples from the SW2 group because those samples had insufficient serum to test again. Thus, we analyzed 159 samples from the other 5 hospitals for NAb against CEV, LACV, SSHV, and TVTV. We also analyzed NAb to CVV, an unrelated bunyavirus that should not be cross-reactive, as a negative control. 

**Figure 4:**
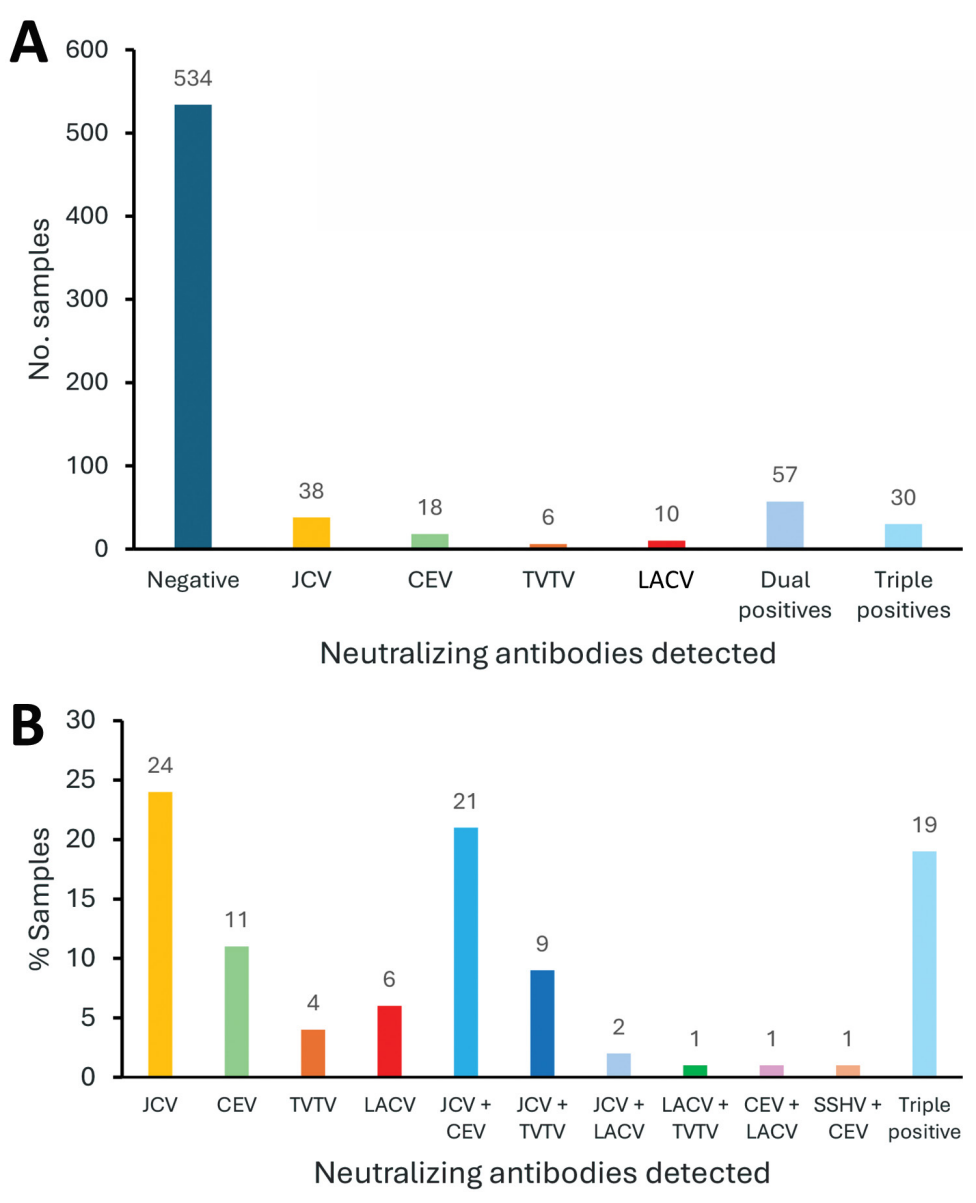
Numbers and percentages of positive samples in a study of neutralizing antibodies against California serogroup orthobunyaviruses in human serum samples, Montana, USA. A) Number of positive and negative samples among 694 tested (excluding samples that could not be analyzed by subsequent screening), 23% of all samples with neutralizing antibody responses. B) Percentage of each virus in 100 samples analyzed from the East region, regardless of their initial positivity to JCV. These samples were analyzed independently for neutralizing antibodies against LACV, CEV, JCV, SSHV, TVTV, and CVV; 60% of positive samples were for positive for JCV or CEV or dual positive for CEV and JCV. CEV, California encephalitis virus; CVV, Cache Valley virus; JCV, Jamestown Canyon virus; LACV, La Crosse virus; SSHV, snowshoe hare virus; TVTV, Trivittatus virus.

We tested 5-fold serial dilutions of serum against each virus. Then, we determined the highest titer of neutralizing activity per virus and considered that virus to be the potential infecting virus (Appendix Table 1). Of the 159 tested samples, 38 (23.8%) had highest titers against JCV compared with other CSG viruses (Table 1, Figure 4). Among the other samples, 18 (11.3%) had the highest neutralizing titers against CEV, 10 (6.2%) against LACV, 6 (3.8%) against TVTV and the other 87 (54.7%) had at least 2 viruses with matching high titers. We observed no high titers for SSHV, despite identification of this virus in Montana (*16*). We did not detect single high NAb titers against CVV. Our results suggest human infections by JCV, CEV, LACV, and TVTV in the state of Montana.

Of 87 samples that had at least 2 viruses with matching high NAb titers, 33 (37.9%) were cross-reactive for JCV and CEV, 14 (16%) for JCV and TVTV, and 3 (3.4%) for JCV and LACV NAbs. Another 30 (34.5%) samples (19% of all samples) had comparable titers for >3 viruses. Thus, we could not determine a single virus agent for over half of the Nab-positive samples, and most of those samples had similar NAb titers for JCV and CEV.

### Cross-Reactivity between JCV and CEV

JCV and CEV had the most samples with cross-reactivity, which was surprising because CEV is more closely related to SSHV and LACV than to JCV (*29*,*30*). However, genetic relatedness does not necessarily directly correlate with NAb cross-reactivity within CSG viruses (*6*). Although the exact neutralizing epitopes for the CSG viruses are not known, the glycoprotein C (GC) head domain is the primary target for orthobunyavirus NAbs (*31*). Therefore, we examined the genetic relationships via amino acid identities in the glycoprotein N (GN), GC, and GC head domain between CSG viruses analyzed in this study. Consistent with previous results (*7*), CEV and TVTV had the lowest percent identities to JCV for GC, GN, and GC head domain (Table 2). CEV and JCV had only 57.6% identity in the GC head domain, suggesting conformational epitopes are likely a factor in NAb cross-reactivity.

**Table 2:** Amino acid similarity among proteins in a study of neutralizing antibodies against California serogroup orthobunyaviruses in human serum samples, Montana, USA*

Virus	% GN/GC similarity				
	LACV	SSHV	CEV	JCV	TVTV
LACV		97.6	95.9	90.5	83.0
SSHV	89.4		95.2	89.8	82.3
CEV	78.2	79.3		89.8	82.7
JCV	68.9	69.7	67.9		81.0
TVTV	65.4	66.1	64.6	63.0	
GC head domain					
SSHV	87.1				
CEV	76.9	78.4			
JCV	57.6	59.2	57.6		
TVTV	62.0	65.1	61.6	54.5	

*GN percent identity is above gray boxes, GC is below gray boxes. CEV, California encephalitis virus; E, east; GC, glycoprotein C; GN, glycoprotein N; JCV, Jamestown Canyon virus; LACV, La Crosse virus; SSHV, snowshoe hare virus; TVTV, Trivittatus virus.

We then assessed whether that cross-reactivity would be observed in mice, which have known inoculating viruses and doses. We inoculated mice with JCV or CEV, collected blood at 21 days postinoculation, and subsequently tested for JCV and CEV NAb titers. 

**Figure 5:**
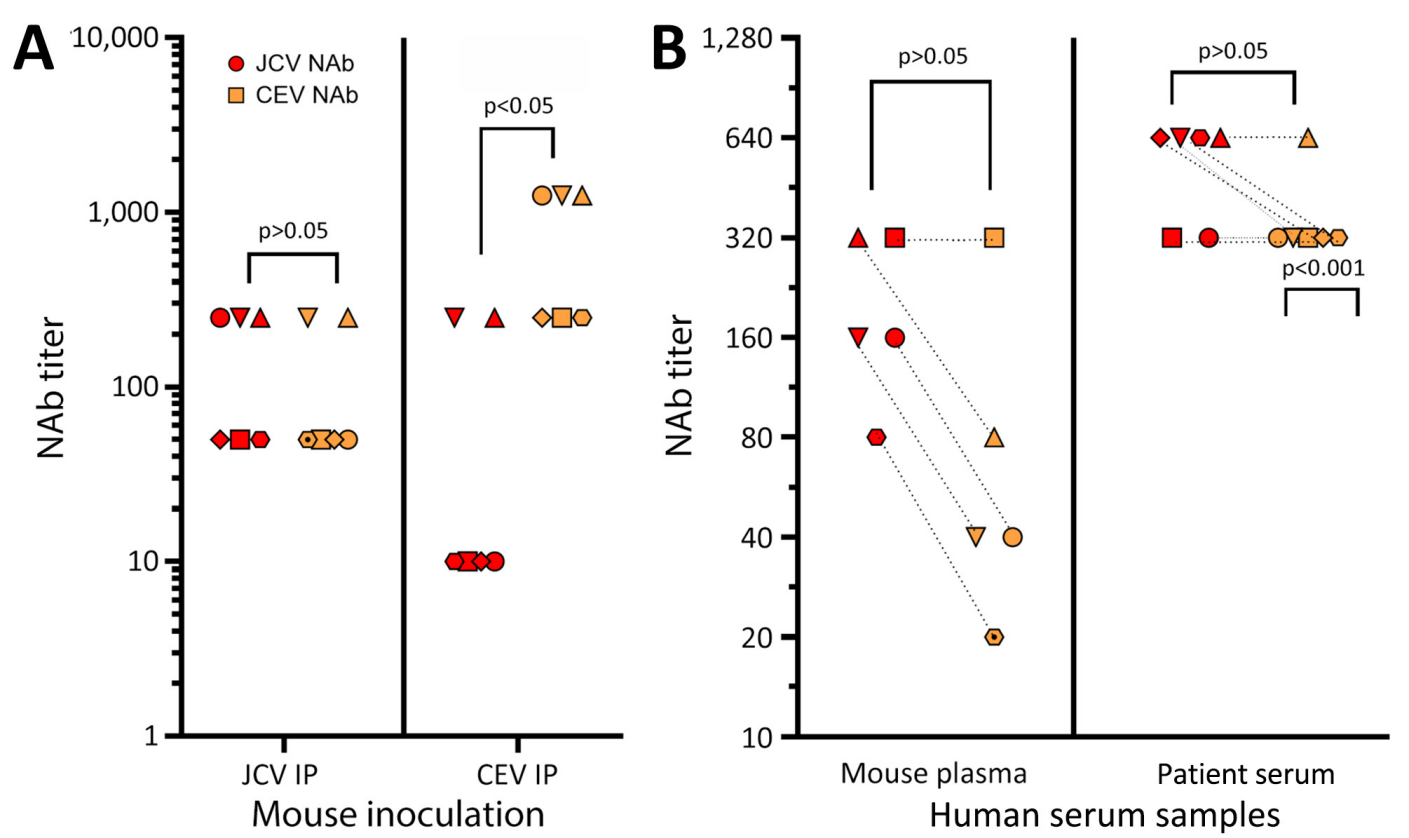
Cross-reactivity between JCV and CEV NAb responses in mice and humans in a study of neutralizing antibodies against California serogroup orthobunyaviruses in human serum samples, Montana, USA. A) Mouse inoculation at 5-fold dilution. Plasma from mice infected with JCV or CEV at 3 weeks postinfection were analyzed for the ability to neutralize JCV or CEV by using 5-fold dilutions of plasma. Each symbol represents an individual mouse with the highest dilution that neutralized 50% of virus. B) Comparison of mouse plasma and human serum samples analyzed at 2-fold dilutions for JCV and CEV responses. Plasma samples were from the same set of mice; sample symbols and dotted lines connect for each virus. Four human serum samples that were dual positive for high titers of JCV and CEV at 1:1,250 (Appendix Figure 1) were analyzed at 2-fold dilution. Samples with p values were at the borderline of 50% inhibition between 1:640 and 1:320. For each set, data were analyzed by a paired t-test for differences in the NAb response between JCV NAb and CEV NAb. CEV, California encephalitis virus; IP, intraperitoneal; JCV, Jamestown Canyon virus; NAb, neutralizing antibodies.

Plasma from JCV-inoculated mice had similar NAb titers for JCV and CEV (Figure 5, panel A), and titers had no statistical difference in paired *t*-test analysis. In contrast, plasma from CEV-inoculated mice significantly inhibited CEV at higher dilutions than inhibition of JCV, by a 5-fold difference (p<0.05). Thus, JCV infection appears to induce antibodies that can neutralize CEV and JCV, but CEV infection does not induce antibodies that neutralize JCV.

To determine whether more precise dilutions would differentiate the NAb response between JCV and CEV, we next used 2-fold, rather than 5-fold, dilutions of plasma from JCV-inoculated mice. Using 2-fold dilutions, we observed a small difference, and JCV NAb titers were higher than CEV NAb titers for most mice (Figure 5, panel B), although not statistically significant. We then chose 5 human serum samples that had a 1:250 NAb titer for both JCV and CEV (Appendix Table 1) and analyzed those by 2-fold dilutions (Figure 5, panel B). We noted a slight, but not statistically significant, difference between JCV and CEV NAbs in human serum, and higher neutralization of JCV in a few, but not all, dual-reactive samples. Thus, for those samples, 2-fold dilutions did not delineate between JCV and CEV.

### CSG NAb Positivity by Sex and Age

We next studied whether we could discern any difference in positive samples within the larger group on the basis of sex or age. Several samples did not have sex or age data and were excluded from this analysis (Appendix Table 1). Of the 159 samples initially positive for JCV and analyzed for cross reactivity, 73 were female patients, 83 were male patients, and 3 samples lacked sex or age data. Among those samples, the median age for female patients was 57 years and median age for male patients was 68 years. We detected positive CSG NAbs in all age groups, except women >80 and years of age and male persons 11–20 years of age (Appendix Figure 1). Of note, we saw no clear distinction in the single positive or dual and triple positive samples across age or sex (Appendix Figure 1). However, observation of NAb responses in almost all ages of both sexes indicate CSG infection is prevalent in Montana.

### Unbiased Screen of Serum Samples from the Eastern Region

**Figure 6:**
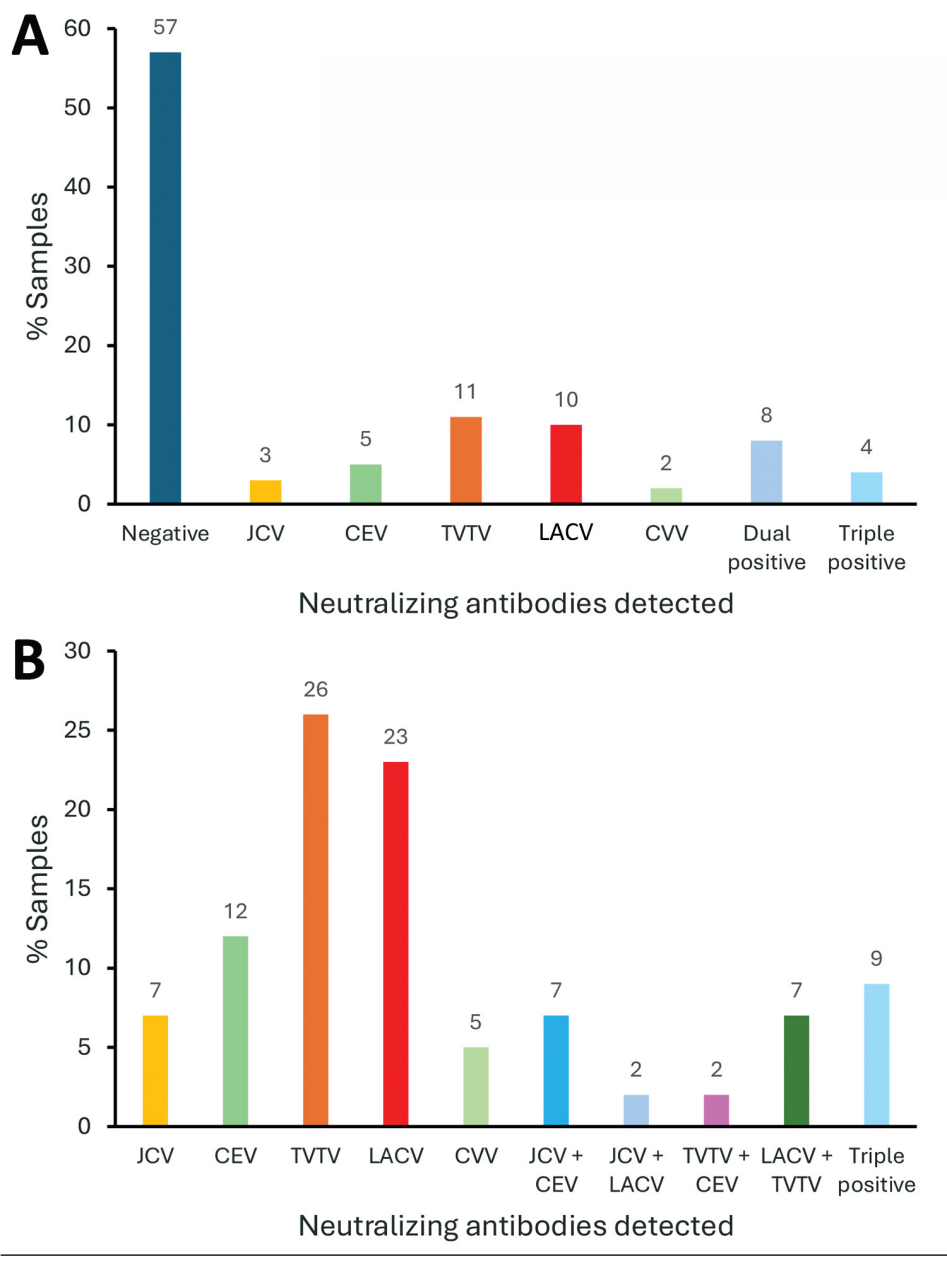
Percentage of positive samples from the east region in a study of neutralizing antibodies against California serogroup orthobunyaviruses in human serum samples, Montana, USA. A) Relative percentage of 100 samples with neutralizing antibodies (Table 3); B) percentage of each virus detected at >1:50 dilution in 43 samples. CEV, California encephalitis virus; CVV, Cache Valley virus; JCV, Jamestown Canyon virus; LACV, La Crosse virus; TVTV, Trivittatus virus.

We initially conducted screening for JCV seropositivity because that was the only CSG with a confirmed case report of encephalitis in Montana. To determine the full potential for CSG infections in Montana, we completed an unbiased screen against all CSG viruses for all serum samples from 1 region. We chose the east region sample set because that group had the highest (31%) positivity rate and the highest number of different viruses detected (Table 1). We analyzed all 100 samples in that group via PRNT for all 6 CSG viruses (Appendix Table 2). Using that unbiased screen, we saw a high number of samples that were positive for TVTV NAb at a 1:10 dilution. Those mock samples for PRNT assay had high background, which made distinguishing that low dilution difficult. We had not observed that in the previous TVTV NAb assay analysis. Due to the limited quantity of serum, we were unable to run all those samples again. To account for the potential background issue in the unbiased screen, we only counted samples as confirmed positive for NAb >50-fold dilution (Table 3, Figure 6). Overall, 40 of 100 samples had NAbs against CSG viruses at a dilution of >50-fold and 3 samples had NAbs against CVV (Table 3, Figure 4). Another 43 samples were NAb positive, but only at a 1:10 dilution, which could be due to background inhibition.

**Table 3:** Unbiased analysis of the 100 samples from east region in a study of neutralizing antibodies against California serogroup orthobunyaviruses in human serum samples, Montana, USA*

Viruses	Neutralizing antibody titer dilution			
	>1,250	250	50	10†
Total no. samples	2	21	20	45
No. single positive‡				
JCV	0	2	1	0
SSHV	0	0	0	0
LACV	0	9	1	0
CEV	1	2	2	2
TVTV	0	2	9	25
CVV	0	0	2	2
No. dual positive§				
JCV + LACV	0	1	0	0
JCV + CEV	1	0	2	1
LACV + TVTV	0	2	1	0
CEV + TVTV	0	0	1	8
TVTV + CVV	0	0	0	4
No. triple positive¶	0	3	1	3

*Highest titer of neutralization >50 for any California serogroup virus. CEV, California encephalitis virus; CVV, Cache Valley virus; E, east; JCV, James Canyon virus; LACV, La Crosse virus; NC, north central; NW, northwest; SC, south central; SSHV, snowshoe hare virus; SW, southwest; TVTV, Trivittatus virus. †Number detected only at 1:10 dilution. ‡Samples that had a 5× titer for 1 specific virus. §Samples with highest titer against 2 viruses. ¶Samples with highest titer against >2 viruses.

In the unbiased analysis, the samples with NAbs against a single CSG had reactivity against TVTV (11%), LACV (10%), CEV (4%), and JCV (3%). The initial JCV screening did pick up the LACV, CEV, and JCV samples, but missed 9 of 11 TVTV-positive samples. NAb to CVV, which unsurprisingly was entirely missed by the JCV screening, was detected in 2% of the unbiased serum specimens. In addition, 16 samples from the unbiased screen were dual or triple positive for NAbs, and 4 of those samples were missed by the initial JCV screen. Thus, the unbiased screen detected a higher prevalence of samples with NAbs against peribunyaviruses found in North America than the prescreen against JCV.

## Discussion

Only 1 case of CSG encephalitis has been reported in Montana, a case of JCV encephalitis originally misdiagnosed as West Nile virus infection (*32*). However, our results suggest a high level of human infection with CSG viruses in Montana; 21%–40% of human serum samples had NAb to >1 CSG (Tables 1,3). Presumably, many of those infections occurred in Montana, although some of samples could be from visitors from another state or residents who acquired infection outside of Montana. Thus, CSG infections in the human population may be prevalent even in areas where limited CSG encephalitis cases have been reported.

Several samples had comparably high NAb titers between CSG viruses (Appendix Table 1). The overlap in NAb cross-reactivity did not correlate strongly with genetic similarity between viruses, and JCV and CEV showed the most cross-reactivity. Reducing dilutions to 2-fold did not clarify CEV and JCV dual-positive samples. However, 2-fold dilutions may distinguish between other dual-reactive viruses such LACV and TVTV, or CEV and TVTV, which we did not test. The high cross-reactivity in human serum samples correlates with cross-reactivity of NAbs to CSG viruses in mice, where the highest NAb titer tested was not always against the inoculating virus and genetic relatedness correlated with NAb cross-reactivity (*6*). That suggests a complex relationship in NAb responses for CSG viruses, in which some viruses generate broader NAbs across multiple CSG viruses. Thus, specific determination of the etiologic CSG for encephalitis cases might be difficult, especially in areas where multiple viruses are present.

In a previous study of national park employees in Wyoming and Colorado, JCV was the primary virus for which NAbs were detected in both states (*12*). However, CEV was not tested. In our study, many of the samples that initially scored as positive for JCV NAb later had an equivalent or higher NAb titer against CEV (Appendix Table 1). Thus, the lower incidence of JCV seropositivity in our study could be due to samples identified as CEV Nab–positive. The high level of cross-reactivity between those 2 viruses and the lack of sequential plasma samples from individual patients prevented us from determining which virus caused the original infection. A retrospective analysis of serum samples from patients identified with JCV encephalitis could help determine whether they also had high NAb titers against CEV.

Although the deidentified nature of this study did not enable us to determine the locations of persons with positive NAb to CSG viruses, we were able to analyze whether age or sex influenced NAb positivity. We did not see any clear correlation with age for detection of NAb between viruses, which is unusual considering that JCV encephalitis cases are primarily observed in adults and LACV encephalitis cases are primarily observed in children <16 years of age (*11*,*20*,*22*). Therefore, the age difference in encephalitis does not appear to be associated with differences in infection, but rather might be due to the differences in the viruses being able to evade immune responses or invade the CNS in adults versus children. Testing for those viruses in cases of viral encephalitis in addition to better surveillance of virus prevalence in mosquitoes may provide clearer insights into the differences in pathogenesis between CSG viruses.

Many of the mosquito vectors and animal hosts for the CSG viruses tested in our study are found in Montana. For example, *Ae. trivittatus*, the main mosquito vector for TVTV, and *Ae. dorsalis*, a primary mosquito vector for CEV, are abundant in Montana (*33*). Although the primary vector for LACV, the *Ae. triseriatus* mosquito, is not in Montana, another mosquito vector, *Ae. canadensis*, is common in the state (*33*,*34*). Multiple overlapping JCV and SSHV vectors are found in Montana (*33*,*35*). Animal reservoirs for CSG also overlap in Montana. CEV, LACV, SSHV, and TVTV infect small rodents including chipmunks and squirrels and CEV, JCV, SSHV, and TVTV have been found in rabbits and snowshoe hares (*17*,*35*–*38*). The overlapping vectors and animal reservoirs in Montana may hinder the process of differentiating between CSG viruses.

The development of new tests, such as the JCV IgM ELISA, may make testing for CSG viruses more accessible. Although defining the cause of viral encephalitis currently does not affect potential treatment, it could drive priorities for the development of new therapeutics and make it easier for virus-focused therapies for encephalitic cases. 

In summary, the prevalence of vector mosquito species and animal reservoirs, as well as the high incidence of human infection in Montana, suggest that CSG viruses should be considered as a potential cause of viral encephalitis cases for northwestern states like Montana. Clinicians should consider CSG viruses in the differential diagnosis for cases of human encephalitis.

Appendix Additional information on neutralizing antibodies against California serogroup Orthobunyaviruses in human serum samples, Montana, USA.
