# Supplementary material for: Neutralizing Antibodies against California Serogroup Orthobunyaviruses in Human Serum Samples, Montana, USA
Source: Emerg Infect Dis. 2025 Apr;31(4):699–709. doi: 10.3201/eid3104.241520 (PMC11950286; doi:10.3201/eid3104.241520)
Supplement: Appendix — Additional information on neutralizing antibodies against California serogroup Orthobunyaviruses in human serum samples, Montana, USA. [file 24-1520-Techapp-s1.pdf]

Article DOI: https://doi.org/10.3201/eid3104.241520

EID cannot ensure accessibility for supplementary materials supplied by authors. Readers who have difficulty accessing supplementary content should contact the authors for assistance.

Neutralizing Antibodies against California Serogroup Orthobunyaviruses in Human Serum Samples, Montana, USA

Appendix

Appendix Table 1. Neutralizing titers per virus for all samples positive for JCV Nabs*

	Neutralizing antibody titer†						
Sample no.	JCV	CEV	LACV	TVTV	SSHV	CVV	Age, y/sex‡
SC 75	1,250	1,250	250	10	250	–	74/M
E 31	1,250	1,250	50	50	250	50	69/M
NW 109	1,250	1,250	50	50	250	–	73//F
NW 107	1,250	1,250	50	10	50	–	67/F
NW 101	1,250	250	50	50	50	–	64/M
NW 166	1,250	50	50	10	50	–	41/M
NW 185	1,250	50	50	10	50	–	62/F
NC 147	1,250	50	10	10	50	–	72/M
SC 45	1,250	50	10	50	10	–	40/F
NW 98	1,250	50	50	10	10	–	57/F
NW 72	250	1,250	1,250	10	1,250	–	52/M
SC 74	250	1,250	250	10	250	–	27/F
NW 142	250	1,250	50	10	250	–	NK/M
NW 88	250	1,250	250	–	50	–	0/M
E 14	250	1,250	10	50	10	–	92/M
E 7	250	250	250	50	50	10	14/F
NC 11	250	250	50	50	50	–	68/M
NC 12	250	250	50	50	50	–	78/M
NW 128	250	250	50	50	50	–	53/F
NC 5	250	250	10	10	50	50	64/M
NW 97	250	250	50	10	50	–	NK/M
E 58	250	50	250	10	50	–	73/M
NW 118	250	250	10	10	50	–	87/M
NW 8	250	50	50	50	50	–	63/F
NC 32	250	50	50	10	50	–	41/M
NW 83	250	50	10	10	10	10	68/M
E 88	250	50	10	10	10	–	48/F
NC 197	250	50	10	10	10	–	55/M
NC 31	250	50	10	10	10	–	73/F
NW 102	250	50	10	10	10	–	58/M
NW 155	250	50	10	10	10	–	28/F
NW 190	250	50	10	10	10	–	54/F
NW 20	250	50	10	10	10	–	73/F
NW 67	250	50	10	10	10	–	75/M
SW 87	250	50	10	10	10	–	37/M
NW 84	250	50	10	–	10	–	61/M
E 11	250	10	–	10	–	10	71/M
NW 106	50	1,250	250	10	250	–	77/M
E 62	50	250	250	50	250	10	85/M
E 44	50	250	250	50	250	–	56/F
SC 61	50	250	250	50	50	–	79/M
NW 175	50	250	250	10	50	–	47/M
E 64	50	10	250	250	10	–	58/M
E 94	50	50	50	250	10	–	79/M

Sample no.	Neutralizing antibody titer† JCV	CEV	LACV	TVTV	SSHV	CVV	Age, y/sex‡
E 49	50	50	250	50	10	–	68/M
E 77	50	250	50	10	10	–	97/M
SC 21C	50	250	10	–	50	–	64/M
E 34	50	250	10	10	10	–	63/M
E 52	50	10	250	10	10	–	92/M
SW 76	50	50	50	50	50	–	65/M
NW 94	50	50	50	10	50	10	NK/M
NW 143	50	50	50	50	10	10	0/M
E 82	50	50	10	50	10	–	66/F
NC 82	50	50	50	10	10	–	70/F
NW 15	50	50	10	–	50	–	80/M
E 90	50	50	10	10	10	10	82/M
E 95	50	50	10	10	10	–	62/M
NC 59	50	50	10	10	10	–	30/M
NC 87	50	50	10	10	10	–	75/F
NW 27	50	50	10	10	10	–	75/M
NW 85	50	50	10	10	10	–	62/F
SC 47	50	50	10	10	10	–	67/F
SW 37	50	50	10	10	10	–	76/M
NW 159	50	10	10	50	10	–	69/F
NC 167	50	50	–	10	10	–	60/F
NW 113	50	50	–	10	10	–	72/F
NC 178	50	50	10	–	10	–	67/M
NW 153	50	50	10	–	10	–	72/M
SC 19C	50	50	10	–	10	–	77/M
SC 59	50	50	10	–	10	–	39/F
SC 79	50	50	10	–	10	–	48/F
SW 12	50	50	10	–	10	–	68/M
NW 71	50	10	10	10	10	–	66/M
SW 6	50	10	10	10	10	–	75/F
SC 63	50	10	10	–	10	–	78/M
SW 69	50	10	10	–	10	–	48/F
SC 65	50	10	–	–	–	–	41/M
SW 93	50	10	–	–	–	–	41/F
E 6	50	–	–	10	–	–	56/M
NC 98	10	1,250	50	10	50	–	47/M
E 9	10	10	250	250	–	10	85/M
NC 99	10	250	50	50	50	–	65/F
SC 77	10	250	50	50	50	–	65/F
SC 60	10	250	50	10	50	–	38/M
E 22	10	50	250	50	10	–	88/M
E 8	10	50	250	50	–	10	55/F
NC 100	10	250	50	–	50	–	53/M
SW 50	10	50	10	250	10	–	80/F
NC 112	10	250	–	10	50	–	78/F
E 17	10	10	250	10	–	–	40/F
E 43	10	10	250	10	–	–	75/M
E 15	10	–	250	10	–	–	45/F
E 21	10	–	250	10	–	–	69/M
E 63	10	–	250	10	–	–	47/F
E 3	10	–	–	250	–	–	27/F
NW 48	10	50	10	10	50	10	41/M
NC 111	10	50	10	–	50	–	93/M
SC 9C	10	50	–	–	50	–	74/M
NW 172	10	50	10	–	10	10	77/M
NW 186	10	10	10	50	10	–	76/F
NC 176	10	50	–	10	10	–	62/M
SW 73	10	10	10	50	–	–	88/M
E 91	10	50	–	–	10	–	75/F
E 57	10	–	50	10	–	–	93/M
SW 95	10	50	–	–	–	–	28/F
NC 56	10	–	–	50	–	–	55/F
NC 62	10	10	10	10	10	–	76/M
NW 183	10	10	10	–	10	10	77/M
SC 52	10	10	10	10	10	–	71/F
SW 51	10	10	–	10	10	–	69/F
NC 171	10	10	–	10	–	10	51/F
NW 76	10	10	–	–	10	–	67/F

Sample no.	Neutralizing antibody titer† JCV	CEV	LACV	TVTV	SSHV	CVV	Age, y/sex‡
SW 9	10	10	–	–	10	–	64/F
E 56	10	10	–	10	–	–	39/F
NC 133	10	10	–	10	–	–	0/F
NC 48	10	10	–	10	–	–	28/F
NW 56	10	10	–	10	–	–	59/F
NW 74	10	10	10	–	–	–	28/F
SW 85	10	10	10	–	–	–	32/F
SW 88	10	10	10	–	–	–	31/F
SW 96	10	10	10	–	–	–	33/F
NW 165	10	–	10	10	–	–	42/F
SW 14	10	–	10	10	–	–	26/M
SW 32	10	–	10	10	–	–	75/F
SW 63	10	–	10	10	–	–	52/F
SW 90	10	–	10	10	–	–	80/M
E 65	10	10	–	–	–	–	58/M
NC 187	10	10	–	–	–	–	79/M
NW 104	10	10	–	–	–	–	70/F
NW 30	10	10	–	–	–	–	78/F
NW 31	10	10	–	–	–	–	74/M
NW 37	10	10	–	–	–	–	76/F
SC 7C	10	10	–	–	–	–	76/M
NC 113	10	–	–	10	–	–	74/F
NC 143	10	–	–	10	–	–	48/M
NC 163	10	–	–	10	–	–	65/F
NC 170	10	–	–	10	–	–	55/F
NC 27	10	–	–	10	–	–	68/F
NC 61	10	–	–	10	–	–	72/M
NC 64	10	–	–	10	–	–	33/F
NW 12	10	–	–	10	–	–	40/M
NW 145	10	–	–	10	–	–	19/F
NW 148	10	–	–	10	–	–	38/M
NW 149	10	–	–	10	–	–	73/F
NW 59	10	–	–	10	–	–	68/F
NW 68	10	–	–	10	–	–	58/M
NC 63	10	–	10	–	–	–	8/M
NW 160	10	–	10	–	–	–	20/F
NC 10	10	–	–	–	–	–	28/M
NC 114	10	–	–	–	–	–	44/F
NC 179	10	–	–	–	–	–	24/F
NW 13	10	–	–	–	–	–	45/F
NW 17	10	–	–	–	–	–	83/M
NW 171	10	–	–	–	–	–	66/M
NW 39	10	–	–	–	–	–	21/M
NW 41	10	–	–	–	–	–	78/F
SC 49	10	–	–	–	–	–	76/M
SW 102	10	–	–	–	–	–	59/M
SW 97	10	–	–	–	–	–	72/M

*De-identified samples were provided with age and sex information and numbered for each group. CEV, California encephalitis virus; CVV, Cache Valley virus; E, East; JCV, James Canyon virus; LACV, La Crosse virus; NC, north central; NW, northwest; SC, south central; SSHV, snowshoe hare virus; SW, south-west; TVTV, Trivittatus virus.
†Highest dilution that yielded 50% reduction of plaques for each virus. The overall highest dilution, suggestive of the inoculating virus is highlighted in blue for single positives, gray for dual positives. Samples are sorted based on titer for JCV, and then overall titers.
‡Age (rounded to the nearest year) of each patient.

Appendix Table 2. Neutralizing titers per virus in unbiased screen of East samples*

Sample no.	Neutralizing antibody titer† JCV	CEV	LACV	TVTV	SSHV	CVV	Age,y/sex‡
E 31	1,250	1,250	50	50	250	50	69/M
E 14	250	1,250	10	50	10	–	92/M
E 7	250	250	250	50	50	10	14/F
E 62	50	250	250	50	250	10	85/M
E 44	50	250	250	50	250	–	56/F
E 58	250	50	250	10	50	–	73/M
E 64	50	10	250	250	10	–	58/M
E 9	10	10	250	250	–	10	85/M
E 49	50	50	250	50	10	–	68/M
E 94	50	50	50	250	10	–	79/M
E 77	50	250	50	10	10	–	97/M
E 22	10	50	250	50	10	–	88/M
E 8	10	50	250	50	–	10	55/F
E 88	250	50	10	10	10	–	48/F
E 52	50	10	250	10	10	–	92/M
E 34	50	250	10	10	10	–	63/M
E 11	250	10	–	10	–	10	71/M
E 17	10	10	250	10	–	–	40/F
E 43	10	10	250	10	–	–	75/M
E 15	10	–	250	10	–	–	45/F
E 21	10	–	250	10	–	–	69/M
E 63	10	–	250	10	–	–	47/F
E 3	10	–	–	250	–	–	27/F
E 82	50	50	10	50	10	–	66/F
E 90	50	50	10	10	10	10	82/M
E 95	50	50	10	10	10	–	62/M
E 99	–	50	10	50	–	10	39/F
E 93	–	10	50	50	–	–	55/F
E 57	10	–	50	10	–	–	93/M
E 91	10	50	–	–	10	–	75/F
E 40	–	10	10	50	–	–	68/F
E 73	–	10	10	50	–	–	60/F
E 92	–	10	10	50	–	–	72/M
E 6	50	–	–	10	–	–	56/M
E 59	–	50	–	10	–	–	37/F
E 2	–	10	–	50	–	–	59/F
E 5	–	10	–	50	–	–	43/F
E 18	–	–	–	50	–	–	67/M
E 28	–	–	–	50	–	–	72/F
E 33	–	–	–	50	–	–	50/M
E 74	–	–	–	50	–	–	30/F
E 100	–	–	–	–	–	50	45/M
E 71	–	–	–	–	–	50	45/M
E 56	10	10	–	10	–	–	39/F
E 13	–	10	10	10	–	–	26/F
E 76	–	10	10	10	–	–	55/F
E 65	10	10	–	–	–	–	58/M
E 1	–	10	–	10	–	–	33/F
E 29	–	10	–	10	–	–	53/F
E 30	–	10	–	10	–	–	73/F
E 35	–	10	–	10	–	–	69/M
E 54	–	10	–	10	–	–	68/M
E 75	–	10	–	10	–	–	67/M
E 79	–	10	–	10	–	–	13/F
E 83	–	10	–	10	–	–	53/F
E 10	–	–	–	10	–	10	39/F
E 32	–	–	–	10	–	10	40/F
E 39	–	–	–	10	–	10	1/F
E 81	–	–	–	10	–	10	48/F
E 36	–	10	–	–	–	–	63/F
E 48	–	10	–	–	–	–	74/M
E 16	–	–	–	10	–	–	30/F
E 19	–	–	–	10	–	–	73/M
E 20	–	–	–	10	–	–	54/M
E 24	–	–	–	10	–	–	64/F
E 25	–	–	–	10	–	–	64/F
E 26	–	–	–	10	–	–	85/M

Sample no.	Neutralizing antibody titer†						Age,y/sex‡
	JCV	CEV	LACV	TVTV	SSHV	CVV	
E 27	–	–	–	10	–	–	25/F
E 4	–	–	–	10	–	–	24/F
E 41	–	–	–	10	–	–	67/M
E 42	–	–	–	10	–	–	35/F
E 45	–	–	–	10	–	–	49/M
E 46	–	–	–	10	–	–	48/F
E 55	–	–	–	10	–	–	34/M
E 66	–	–	–	10	–	–	35/M
E 67	–	–	–	10	–	–	46/F
E 69	–	–	–	10	–	–	68/M
E 72	–	–	–	10	–	–	93/F
E 80	–	–	–	10	–	–	18/M
E 84	–	–	–	10	–	–	64/F
E 85	–	–	–	10	–	–	73/F
E 86	–	–	–	10	–	–	69/F
E 87	–	–	–	10	–	–	58/F
E 96	–	–	–	10	–	–	62/M
E 97	–	–	–	10	–	–	86/F
E 98	–	–	–	10	–	–	40/M
E 12	–	–	–	–	–	10	25/F
E 61	–	–	–	–	–	10	79/NK

*De-identified samples from the East region were tested against all viruses for NAb titer, regardless of positivity toward JCV NAb. Samples are sorted based on overall NAb cross-reactivity. CEV, California encephalitis virus; CVV, Cache Valley virus; E, East; JCV, James Canyon virus; LACV, La Crosse virus; SSHV, snowshoe hare virus; TVTV, Trivittatus virus; –, not detected.

†Highest dilution that yielded 50% reduction of plaques for each virus. The overall highest dilution, suggestive of the inoculating virus is highlighted in blue for single positives, gray for dual positives. Samples are sorted based on titer for JCV, and then overall titers.

‡Age (rounded to the nearest year) of each patient.

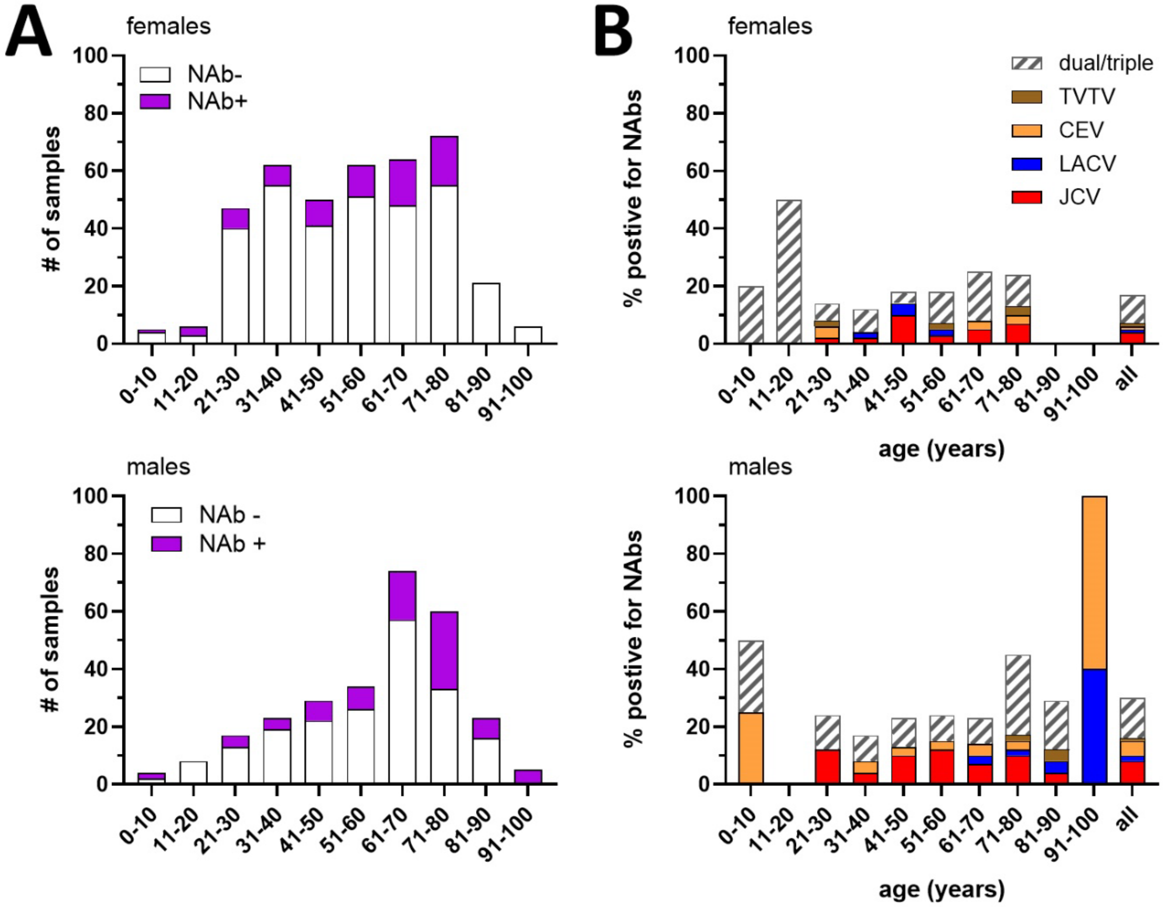

Appendix Figure. Human serum samples from Table 1 were analyzed on the basis of age and sex. A) Initial JCV-scored NAb-positive samples relative to JCV-negative samples shown as number of samples for each category. Samples without sex or age data were excluded. B) Breakdown of samples by CSG virus in age and sex categories as a ratio of positive samples for each age category.
